# Chromogranin a gene variants influence survival at old age through pleiotropic effects on multiple age-related traits

**DOI:** 10.3389/fragi.2025.1625070

**Published:** 2025-09-12

**Authors:** Paolina Crocco, Rossella La Grotta, Francesco De Rango, Giuseppe Passarino, Serena Dato, Giuseppina Rose

**Affiliations:** Department of Biology, Ecology and Earth Sciences, University of Calabria, Rende, Italy

**Keywords:** Chromogranin A, aging, longevity, age-related diseases, SNP, TyG index, MMSE, cvd

## Abstract

Aging, age-related diseases, and longevity are interconnected processes influenced by shared molecular and genetic mechanisms. In this study, we investigated the role of genetic variation in the Chromogranin A (*CHGA*) gene, which encodes a multifunctional precursor of regulatory peptides, in human longevity and age-related traits. Using a case-control design with two age cohorts (older adults: 65–85 years; long-lived: 86–107 years), we analysed nine selected *CHGA* single nucleotide polymorphisms (SNPs) for associations with survival to advanced age and relevant clinical parameters. Five SNPs (rs9658628, rs9658631, rs9658634, rs7159323, and rs7610) were significantly associated with longevity (FDR q < 0.05). In the older adult cohort, the 5′-UTR rs9658628-A allele was associated to reduced odds of reaching advanced age and correlated with increased insulin resistance (TyG index), type 2 diabetes, and lower cognitive performance (MMSE scores), traits typically linked to higher mortality risk. Paradoxically, this allele was also associated with a lower risk of cardiovascular disease, suggesting pleiotropic effects potentially mediated by its regulatory effects on *CHGA* expression across different tissues. Functional annotation supported rs9658628 as an expression quantitative trait locus (eQTL) for *CHGA* and neighboring genes (*ITPK1*, *FBLN5* genes in particular) in relevant tissues. Additionally, the 3′-UTR rs7610-T allele was associated with both increased diastolic blood pressure and enhanced survival, highlighting the complexity of blood pressure regulation in aging. Although statistical significance for clinical trait associations was lost after FDR correction, these findings suggest that genetic variations in CHGA exert a complex and multifactorial influence on pathways related to metabolism, cognition, and vascular health, with possible consequences for longevity. This intricate pattern could be due to the multiple, sometimes opposing, functions of CHGA and its active fragments. The biological rationale and potential clinical implications of these associations call for further investigation and independent confirmation.

## 1 Introduction

Chromogranin A (CHGA) is a soluble glycoprotein comprising 439 amino acids and is a member of the granin family—acidic secretory proteins stored in the secretory granules of endocrine, neuronal, and neuroendocrine cells (Taupenot et al., 2003). Over the past decades, CHGA has gathered significant attention for its critical role in maintaining cellular and systemic homeostasis.

Intracellularly, CHGA is essential for the formation of catecholamine-containing secretory vesicles, the trafficking of proteins involved in regulated secretion, and the maintenance of intracellular calcium homeostasis (Bartolomucci et al., 2011). Extracellularly, CHGA functions as a prohormone, whose proteolytic cleavage at its dibasic sites gives rise to multiple biologically active peptides, such as vasostatin, pancreastatin, parastatin, catestatin, and serpinin (see Supplementary Figure S1). These peptides exert pleiotropic effects on diverse physiological processes via autocrine, paracrine, and endocrine mechanisms (Helle et al., 2007; D'Amico et al., 2014).

Numerous *in vitro* and *in vivo* studies, in cellular models, mouse models, and humans, have demonstrated that changes in CHGA expression influence key physiological pathways, including catecholamine secretion, energy metabolism, cholesterol homeostasis, and both vesicular and mitochondrial function (Pasqua et al., 2016; Wollam et al., 2017; Iyer et al., 2023). Moreover, CHGA-derived peptides contribute to host defense and play roles in the acute-phase response (Helle et al., 2018). Importantly, while some of these peptides share regulatory roles, others exert opposing effects. For example, pancreastatin promotes inflammation and impairs insulin sensitivity and glucose tolerance, whereas catestatin has anti-inflammatory properties and counteracts adiposity and hypertension (Bandyopadhyay et al., 2015; Bandyopadhyay and Mahata, 2017). This underscores the significance of CHGA processing in maintaining physiological balance: dysregulation in the production or balance of these bioactive fragments can contribute to disease pathogenesis.

Given their diverse biological functions, CHGA and its derived peptides have been implicated in a wide array of diseases, including cardiovascular conditions, hypertension, type 2 diabetes, cancer, and chronic inflammatory disorders (Helle et al., 2007; D’Amico et al., 2014; Tota et al., 2014; Corti et al., 2018; Mahata and Corti, 2019; Watanabe, 2021; Herold et al., 2018; Zalewska et al., 2022).

*CHGA* is encoded by a highly polymorphic gene located on chromosome 14q32.12. Several population studies have reported associations between *CHGA* genetic variants and susceptibility to age-related diseases such as hypertension, coronary artery disease, heart failure, and metabolic dysfunctions (Subramanian et al., 2017; Allu et al., 2022; Iyer et al., 2023; Marjani et al., 2024). These findings raise the possibility that *CHGA* genetic variation may also influence broader aging-related phenotypes, including longevity.

Based on this background, we hypothesized that genetic variation in *CHGA* may affect the likelihood of reaching advanced age by modulating individual susceptibility to metabolic, cognitive, and cardiovascular traits that influence survival. To test this hypothesis, we analysed 12 SNPs across the *CHGA* locus for association with longevity in a cohort of individuals aged 65–107 years. For SNPs significantly associated with the chance of long life, we further explored their relationships with metabolic parameters, cardiovascular traits, cognitive performance, and multimorbidity indices, aiming to identify possible mediators of their effects on survival.

## 2 Materials and methods

### 2.1 Study subjects

The cohort of this study included 484 individuals aged 65–107 years (mean age 83.68 ± 9.56 years), consisting of 312 females (mean age 84.07 ± 9.45) and 172 males (mean age 82.98 ± 9.77). The samples were obtained from elderly individuals residing in nursing homes across the Calabria region in southern Italy. This was part of a broader investigation into the quality of aging in the entire region. Subjects were eligible to participate in the study if they were of Calabrian ancestry. Socio-demographic information and health details, including medical history and medication use, were gathered through a standardized questionnaire, medical visits, and clinical exams. Fasting venous blood was collected from each participant for clinical testing, laboratory analysis, and DNA extraction.

The study conforms to the Declaration of Helsinki regarding research involving human subjects and the protocol was approved by the local Ethical Committee (Comitato Etico Regione Calabria-Sezione Area Nord) on 2017-10-31 (code n. 25/2017). Each subject signed an informed consent for the permission to collect blood samples and usage of register-based information for research purposes.

### 2.2 Assessment of anthropometric, laboratory and clinical variables

Trained nurses measured participants’ waist and hip circumferences, from which the waist-to-hip ratio (WHR) was calculated. Height and weight were recorded with participants wearing light clothing and no shoes, and body mass index (BMI) was calculated as weight (kg) divided by height squared (m^2^). Blood pressure (BP), including systolic (SBP) and diastolic values (DBP), was measured three times on the right arm using a mercury sphygmomanometer after a rest period of at least 5 min. The average of the three readings was used as the final BP value. Routine hematological and biochemical markers, reported in Table 1, were analysed at the Italian National Research Center on Aging (Cosenza) using standardized methods. The triglyceride-glucose (TyG) index was calculated as Ln [fasting triglycerides (mg/dL) × fasting plasma glucose (mg/dL)/2], following the method by Simental-Mendía et al. (2008). The glomerular filtration rate (GFR) was estimated using the creatinine-based Berlin Initiative Study 1 (BIS1) equation, specifically developed for older adults (Schaeffner et al., 2012). The formula used was: eGFR BIS1 = 3,736 × creatinine^−0.87^ × age^−0.95^ × 0.82 (if female).

**TABLE 1:** Baseline demographic and clinical characteristics of the analysed cohort by age group membership.

Variables	Older adults	Long-lived	P-value^a^
N (age range, years)	269 (65–85)	215 (86–107)	
Age, [Mean (SD)]	76.77 (6.3)	92.33 (4.6)	<0.001
Sex (men, %)	36.8%	34.0%	ns
Anthropometric traits [Mean (SD)]			
BMI	27.45 (5.86)	23.80 (5.22)	<0.001
WC, cm	98.26 (13.50)	91.15 (14.52)	<0.001
HC, cm	105.84 (13.24)	99.68 (15.01)	<0.001
WHR	0.93 (0.08)	0.91 (0.07)	ns
Clinical data [Mean (SD)]			
SBP, mmHg	130.69 (14.33)	131.82 (14.84)	ns
DBP, mmHg	74.37 (10.57)	75.12 (10.05)	ns
TG, mg/dL	126.55 (64.60)	112.16 (51.08)	<0.001
HDL-C, mg/dL	51.84 (20.13)	54.74 (15.21)	ns
LDL-C, mg/dL	99.29 (36.87)	101.30 (36.67)	ns
TC, mg/dL	177.13 (50.88)	181.34 (42.61)	ns
FPG, mg/dL	105.48 (39.32)	98.71 (37.08)	<0.001
HbA1c, %	6.10 (1.58)	5.50 (2.78)	<0.001
TyG index	4.67 (0.31)	4.59 (0.27)	<0.001
Albumin (g/dL)	54.45 (6.72)	53.55 (6.28)	ns
Total protein (g/dL)	6.61 (0.57)	6.60 (0.69)	ns
Creatinine (mg/dL)	1.10 (0.51)	1.07 (0.39)	ns
eGFR-BIS1	54.67 (16.75)	45.80 (13.29)	<0.001
Uric acid (mg/dL)	4.89 (2.81)	5.69 (6.28)	ns
Ferritin (ng/mL)	164.05 (215.7)	192.49 (218.51)	ns
Alkaline Phosphatase (U/L)	158.01 (95.14)	152.27 (103.21)	ns
Total bilirubin, mg/dL	0.66 (0.32)	0.67 (0.31)	ns
C-Reactive Protein (mg/L)	13.88 (42.42)	18.50 (30.60)	0.014
Morbidities			
Cognitive and physical impairment			
*MMSE score*	20.02 (6.39)	13.73 (6.25)	<0.001
*ADL score*	2.83 (2.01)	1.9 (1.7)	<0.001
*HG strength*	21.36 (10.21)	12.59 (6.73)	<0.001
Diabetes (Yes, %)	27.1%	13.7%	<0.001
Hypertension (Yes, %)	71.0%	68.4%	ns
Cardiovascular diseases (Yes, %)	36.8%	60.0%	<0.001
Chronic kidney disease (Yes, %)	64.4%	85.9%	<0.001
CIRS score	14.91 (13.88)	19.69 (14.21)	<0.001

Continuous variables are expressed as mean and standard deviation (SD), while categorical variables are expressed as percentage (%).

^a^ P value from t-test or Mann–Whitney depending on continuous data distribution and from chi-squared test of association for categorical variables.

Abbreviations: BMI, body mass index; WC, waist circumference; HC, hip circumference; WHR, Waist-to-Hip Ratio; SBP, systolic blood pressure; DBP, diastolic blood pressure; TG, triglycerides; HDL-C, High-Density Lipoprotein Cholesterol; LDL-C, Low-Density Lipoprotein Cholesterol; TC, total cholesterol; FPG, fasting plasma glucose; HbA1c, Glycosylated haemoglobin; TyG, Triglyceride-Glucose; eGFR, estimated Glomerular Filtration; MMSE, Mini-Mental State Examination; ADL, activities of daily living; HG, hand grip; CIRS, cumulative illness rating scale.

Hypertension was defined as SBP/DBP ≥140/90 mmHg or use of antihypertensive medication), diabetes as fasting plasma glucose >125 mg/dL or use of antidiabetic therapy, and the presence of cardiovascular disease (CVD) based on a comprehensive review of medical history, clinical symptoms, imaging results, and physical or laboratory examinations, evaluated by a board-certified cardiologist in accordance with international clinical guidelines. Chronic kidney disease (CKD) was defined as an estimated glomerular filtration rate (eGFR) <60 mL/min/1.73 m^2^ using the BIS1 equation.

Multimorbidity was assessed using the modified Cumulative Illness Rating Scale (CIRS), which evaluates the burden of chronic conditions across 14 physiological systems (cardiac, vascular, respiratory, gastrointestinal, hepatic, renal, genitourinary, musculoskeletal, dermatologic, neurologic, endocrine, metabolic, breast, and psychiatric). Each system was scored from 0 (no issue) to 4 (severe impairment), with total scores ranging from 0 to 56. Higher scores indicate greater overall morbidity.

### 2.3 SNPs selection and genotyping

Twelve SNPs encompassing the CHGA gene and its 5′and 3′flanking regions were genotyped in all subjects included in the study. The SNPs were selected from previous studies in which significant genetic associations with cardio-metabolic diseases and/or associated endophenotypes were reported.

Schematic representation of the human chromogranin A gene structure and its derived peptides, as well as the SNPs that were genotyped, are shown in Supplementary Figure S1.

DNA was extracted from whole blood according to standardized procedures. Multiplex SNP genotyping was carried out using PCR, followed by primer extension and MALDI-TOF mass spectrometry with iPLEX Gold technology from Sequenom (Sequenom Inc., San Diego, CA, United States). PCR and single base extension primers were designed using Sequenom MassARRAY Assay Designer software (version 3). PCR amplification was performed using standard methods, and unincorporated nucleotides were removed using shrimp alkaline phosphatase (SAP). A primer extension reaction was then conducted with a mass extension primer and terminator. The extension products were desalted on resin and spotted onto the 384-element SpectroCHIP (Sequenom) for MALDI-TOF analysis, utilizing SpectroACQUIRE v3.3.1.3 software (Sequenom). Data were analyzed with MassARRAY Typer v3.4 Software (Sequenom).

To ensure quality control, approximately 10% of the samples were re-genotyped to verify the accuracy of the genotype identification process, with duplicate concordance exceeding 99.8% for all SNPs. Additional quality control steps included excluding SNPs that were not in Hardy–Weinberg equilibrium in controls (p-value <0.05) or had low genotyping success (<90%).

### 2.4 Functional annotation

*CHGA* gene polymorphisms significantly associated with the phenotypes measured in our study were a subject for comprehensive bioinformatics analysis. In particular, bioinformatics tools of the quantitative trait loci (QTL) databases such as the GTEx portal (https://gtexportal.org) (The GTEx Consortium, 2020), and QTLbase2 (http://mulinlab.tmu.edu.cn/qtlbase) (Huang et al., 2023), were used to evaluate whether the *CHGA* gene polymorphisms represent significant QTLs correlating with a variation of molecular traits such as mRNA expression (eQTL), methylation (mQTL), histone modification (hQTL) and splicing events (sQTL).

The GTEx portal serves as an extensive public resource for researching gene expression and regulation specific to different tissues. QTLbase2 is a database that gathers and compiles genome-wide QTL summary data for various human molecular traits across more than 70 different tissues and cell types. The functionality of these SNPs was further explored by FuncPred (https://snpinfo.niehs.nih.gov/snpinfo/snpfunc.html) (Xu and Taylor, 2009) and Regulome DB (http://regulomedb.org/) (Boyle et al., 2012), which annotate and prioritize potential regulatory variants in the human genome using a collection of functional information gathered from a variety of tools and resources.

### 2.5 Statistics

Analyses were performed by dividing the sample into older adults (269 subjects, 65–85 years; median 76.77 ± 6.3) and long-lived (215 subjects, 86–107 years; 92.33 ± 4.6). Continuous variables are presented as means with standard deviations. To assess data distribution normality, the Kolmogorov-Smirnov test was employed. Where necessary, variables were log-transformed to meet normality assumptions before analysis. The independent-samples t-test or the Mann-Whitney U test, depending on the distribution characteristics, were used to compare differences between groups for continuous variables. Categorical variables are expressed as percentages and were compared using the chi-squared (χ2) test. Hardy-Weinberg equilibrium for each SNP was evaluated in controls using the χ2 test.

Logistic regression models were used to estimate the impact of genotypes on the chance to reach a very advanced age. Genetic data were coded with respect to a dominant, a recessive, and an additive model of inheritance. Then, for each SNP, the most likely genetic model was estimated based on minimum level of statistical significance (Wald test p-value). For SNPs with minor allele homozygotes <3%, either in the group older adults or long-lived group, only the dominant model was considered. In such models, sex was used as a covariate.

Multivariate linear and logistic regression models were used to calculate the association of genetic variants with continuous and dichotomized traits, respectively, adjusting for age, sex, and BMI. SNP × sex interaction terms were included in logistic regression models, adjusting for age. The false discovery rate (FDR) was calculated to correct for multiple comparisons of genotypes, and FDR <0.05 was considered to indicate a statistical significance for association (Benjamini and Hochberg, 1995). Complementary validation analyses included (i) the use of the TSI population from the 1000 Genomes Project (www.1000genomes.org) (n = 114; 59 males and 55 females), limited to allele frequencies and age-association given the absence of phenotypic data, and (ii) a stratified bootstrap approach (Bias-Corrected and Accelerated method, 5,000 resamples, stratified by sex) to internally assess the robustness of the observed associations.

Linkage disequilibrium (LD) and haplotype analysis were conducted using the SHEsis software (Shi and He, 2005).

Statistical analyses were performed using SPSS software (version 29.0, SPSS Inc., Chicago, IL, United States).

## 3 Results

### 3.1 Participant characteristics

A total of 269 older adults (ages 65-85) and 215 oldest-old individuals (ages 86-107) participated in this study. Summary statistics for the anthropometric, biochemical, and clinical variables for both age groups are presented in Table 1. In brief, no significant differences were observed in sex distribution, with a sex ratio imbalance favouring females in both groups. As shown in Table 1, significant differences between the groups were observed in some anthropometric and quantitative metabolic variables, as well as in the presence of morbidities.

### 3.2 Genetic association between *CHGA* gene polymorphisms and survival at old age

**FIGURE 1:**
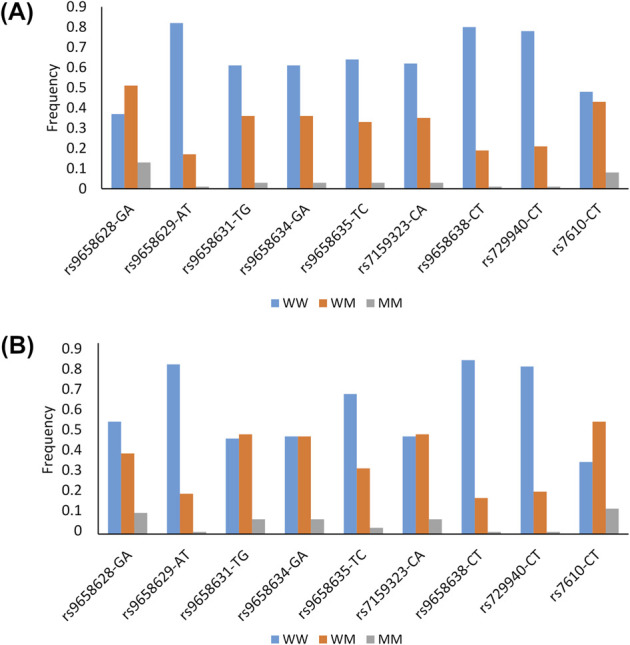
Genotypes frequencies of the *CHGA* polymorphisms in the group of older adults **(A)** and in the group of long-lived subjects **(B)** W: Wild type allele; M: Mutant allele.

Twelve SNPs were selected for this study, and details of their gene position, functional annotation and minor allele frequency (MAF) observed in our samples and in the 1,000 Genomes TSI population (Tuscany, Italy) can be found in Supplementary Table S1. Two of them (rs9658630 and rs9658667) were excluded from the analysis because of low genotyping success rates (<90%), and one (rs9658664) because it deviated from HWE (P < 0.05). The remaining nine SNPs successfully passed the quality control procedures. The genotypic frequencies of these SNPs in each of the two age groups are shown in Figure 1.

Table 2 shows the results of the logistic regression analyses adjusting for sex on the probability of reaching advanced age. Five out of the nine polymorphisms (namely, rs9658628, rs9658631, rs9658634, rs7159323 and rs7610) exhibited a statistically significant association under a dominant genetic model. It emerged that the rs9658628-A allele was associated with a diminished probability of belonging to the oldest age group, with an adjusted odds ratio (OR) of 0.51 (95% CI: 0.35–0.75; p = 0.0005), signifying that the presence of the minor A allele is associated with diminished survival at advanced age. Also, individuals carrying the minor allele at rs9658631-(G), rs9658634-(A), rs7159323-(A), and rs7610-(T) had a higher chance of being in the oldest age group. The adjusted ORs were 1.86 (1.29–2.68), 1.79 (1.24–2.58), 1.88 (1.30–2.71), and 1.80 (1.24–2.62), with p-values of 0.0008, 0.0017, 0.0007, and 0.002, respectively. All the associations remained significant after adjusting for multiple testing with false discovery rate (FDR) q < 0.05. To strengthen the robustness of our results, we performed an additional sensitivity analysis using a Bias-Corrected and Accelerated (BCa) bootstrap analysis with 5,000 resamples, stratified by sex, which confirmed the stability of the observed SNP associations with longevity (results reported in Table 2). In a separate analysis, we tested for sex × SNP interactions (Table 2) and found no significant interactions, indicating that the associations were consistent across sexes.

**TABLE 2:** Results of multivariate logistic regression analysis comparing older adults with long-lived individuals.

SNPs	Older adult vs. long-lived			Bootstrap^c^		SNP × sex interaction
	OR (95% CI)	p-value^a^	FDR^b^	BCa 95% CI	p-value	p-value
rs9658628-G/A	0.51 (0.35–0.75)	0.0005	0.0024	−1.02–-0.28	<0.001	0.65
rs9658629- A/T^d^	1.11 (0.70–1.78)	0.65	0.81	-	-	-
rs9658631- T/G	1.86 (1.29–2.68)	0.0008	0.0024	0.25–0.99	<0.001	0.47
rs9658634- G/A	1.79 (1.24–2.58)	0.0017	0.0036	0.20–0.97	0.003	0.57
rs9658635- T/C	0.95 (0.64–1.39)	0.78	0.81	-	-	-
rs7159323- C/A	1.88 (1.30–2.71)	0.0007	0.0024	0.25–1.01	<0.001	0.33
rs9658638- C/T^d^	0.90 (0.57–1.43)	0.65	0.81	-	-	-
rs729940- C/T^d^	0.95 (0.61–1.48)	0.81	0.81	-	-	-
rs7610- C/T	1.80 (1.24–2.62)	0.002	0.0036	0.24–0.98	0.001	0.61

OR, odds ratio; CI, 95% confidence interval; FDR, false discovery rate.

^a^ All p-values are from the dominant model (best fit) adjusted for sex.

^b^ False discovery rate (FDR) adjusted p-values.

^c^ Bootstrap results are based on 5,000 bootstrap samples; BCa, Bias Corrected and accelerated bootstrap confidence intervals.

^d^ For these SNPs, only the dominant model was considered since the rare homozygous genotype was <3%.

The p-value for the SNP × sex interaction is also reported.

In further support of these findings, the same associations were replicated using a cohort of adult Italian volunteers (Tuscans; TSI; 114 individuals, 59 males and 55 females), whose genotypes for the relevant SNPs were obtained from the 1000 Genomes Project and used as controls for comparison with long-lived individuals. The ORs were 0.58 (0.36–0.93), 1.92 (1.19–3.10), 1.86 (1.15–3.00), 1.85 (1.14–2.98), 2.10 (1.30–3.40), respectively for rs9658628, rs9658631, rs9658634, rs7159323 and rs7610. No association was observed when comparing our sample of older people with the group of Tuscans, suggesting that these SNPs may not differ significantly in the general older population.

**FIGURE 2:**
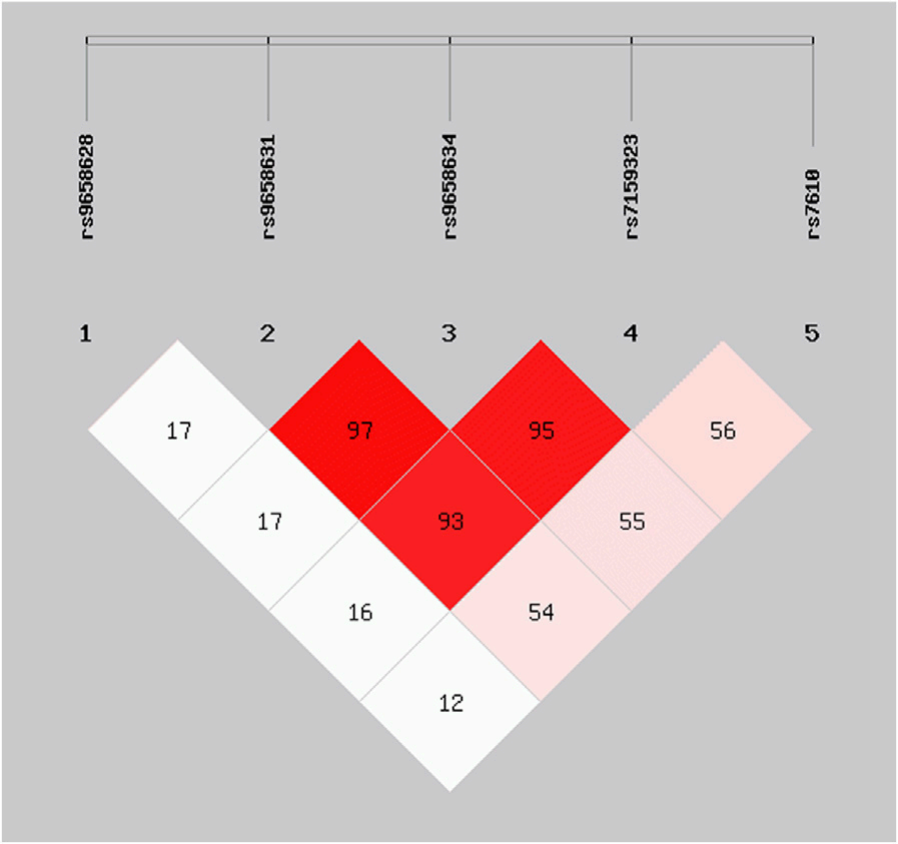
Linkage disequilibrium matrix of the *CHGA* SNPs significantly associated with survival at old age in the analysed cohort. Numerical values shown correspond to r^2^ values.

For identifying independent variants in *CHGA* associated with advanced ages, the LD patterns (r2) of five significant SNPs were examined and depicted in Figure 2. A strong LD was detected among the variants rs9658631, rs9658634, and rs7159323 (r^2^ > 0.9), suggesting that the associations observed at the individual SNPs are not independent and likely reflect a single association signal influencing longevity. The haplotype analysis for these polymorphic sites revealed that haplotype composed of the minor alleles of the three variants (G-A-A) was associated with a significantly increased chance of reaching old age (OR 1.67, CI: 1.24–2.25, p = 0.0006). Conversely, the haplotype containing the major alleles (T-G-C) appeared to have an opposite effect, with an odds ratio of 0.59 (CI: 0.44–0.80, p = 0.0006) (Table 3). Thus, haplotype analysis confirmed association results with the single markers.

**TABLE 3:** Estimated haplotype frequencies defined by *CHGA* SNPs in linkage disequilibrium and their association with survival at old age.

Haplotypes			Frequencies			
rs9658631	rs9658634	rs7159323	Older adults	Long-lived	OR (95% CI)	p-value
T	G	C	0.784	0.685	0.59 (0.44–0.80)	0.0006
G	A	A	0.205	0.299	1.67 (1.24–2.25)	0.0006

Frequencies are shown for older adults and long-lived individuals, along with odds ratios (OR), 95% confidence intervals (CI), and p-values from association analysis.

### 3.3 Association of SNPs with clinical parameters and health conditions

Next, we aimed to investigate whether the five variants associated with advanced age were also associated with clinical parameters and health conditions that are established risk factors for age-related diseases that contribute to significant morbidity and mortality in the elderly. We focused on the age group 64–85 years and assessed the variability of each SNPs in relation to the parameters listed in Table 1. The significant results (p < 0.05), adjusted for age, sex, and BMI, are presented in Table 4. To assess the robustness of these findings, we also performed BCa bootstrap analyses and tested for sex × SNP interactions, which showed that the associations remained consistent and that no significant interactions were present (Table 4).

**TABLE 4:** Significant associations of *CHGA* SNPs with clinical traits in the older adult cohort.

SNP	Parameter	B (SE) or OR (95% CI)	p-value	Bootstrap^a^		SNP × sex interaction
				BCa 95% C	p-value	p-value
rs9658628	TyG	0.10 (0.04)	0.019	0.02–0.19	0.019	0.44
	MMSE	−2.30 (0.83)	0.006	−3.90–−0.60	0.007	0.72
	Diabetes	1.93 (1.02–3.66)	0.038	0.03–1.98	0.035	0.48
	CVD	0.46 (0.24–0.87)	0.017	−1.42–−0.22	0.015	0.53
rs7610	DBP	2.92 (1.23)	0.019	0.39–5.39	0.02	0.63

Results are from linear and logistic regressions adjusted for sex, age, and BMI. Shown are unstandardized coefficients (B) and standard error (SE) or odds ratios (OR) and 95% confidence interval (CI) with corresponding p-values.

^a^ Bootstrap results are based on 5,000 bootstrap samples; BCa, Bias Corrected and accelerated bootstrap confidence intervals.

The p-value for the SNP × sex interaction is also reported.

Abbreviations: TyG, Triglyceride-glucose index; MMSE, Mini-Mental State Examination; DBP, diastolic blood pressure; CVD, cardiovascular disease.

The table shows that the presence of the minor rs9658628-A allele, which was negatively correlated with survival after 85 years of age, was also associated with an increased triglyceride glucose (TyG) index (β = 0.104; p = 0.019), a well-established marker of insulin resistance. Additionally, individuals carrying the rs9658628-A allele had a significantly higher likelihood of having diabetes compared to non-diabetics (OR = 1.93, 95% CI: 1.02–3.66; p = 0.038). Notably, carriers of this allele also scored significantly lower on the Mini-Mental State Examination (MMSE) than non-carriers (β = −2.30; p = 0.006), indicating poorer cognitive performance. Conversely, the rs9658628-A allele appeared to confer a protective effect against cardiovascular disease (CVD), with carriers showing a significantly lower risk compared to non-carriers (OR = 0.46, 95% CI: 0.24–0.87; p = 0.017). The analysis also showed that carriers of the T minor allele of rs7610, who were more likely to live to an older age, had significantly higher diastolic blood pressure (DBP) compared with non-carriers (beta = 2.92; p = 0.019). There was also a marginal, but not significant (p = 0.057) trend towards higher systolic blood pressure (SBP).

Regarding the three SNPs in strong LD (rs9658631, rs9658634, rs7159323), no significant associations were observed with the clinical parameters tested. A marginal effect (p = 0.06) was observed towards an association with higher LDL-C levels.

However, the associations that reached nominal significance did not remain significant after FDR correction for multiple testing.

### 3.4 Functional characterization of variants

We conducted a comprehensive functional annotation of the associated SNPs using integrative multi-omics databases. Supplementary Table S2 provides a synthesis of annotations specifically related to the impact of these SNPs on the *CHGA* gene, while Supplementary Table S3 presents QTLbase-derived annotations referring also to effects on other genes.

Regulatory information was obtained through RegulomeDB and FuncPred. All variants were predicted to have regulatory functions and affect putative transcription factor-binding sites.

To assess transcriptional effects, we examined the expression quantitative trait loci (eQTLs) using the GTEx portal and QTLbase. According to GTEx data, the rs9658628-G allele significantly reduced *CHGA* mRNA levels in the cerebellum, while the rs7610-T allele was associated with increased *CHGA* expression in the pituitary. The rs7610-T allele also correlated with reduced *ITPK1* expression in the aorta and tibial arteries, and lower *LINCO2287* levels in the cerebellum. In contrast, no significant effect on *CHGA* expression across tissues was observed for the linked variants rs9658631, rs9658634, and rs7159323. However, their minor alleles were associated with increased *RIN3* (rs9658631, rs9658634, rs7159323) and *ITPK1* (rs7159323) expression in the aorta.

QTLbase further supported and expanded these findings (see Supplementary Table S2, 3). For rs9658628, additional effects were noted in brain tissues, including altered expression of CHGA in the prefrontal cortex and *ITPK1* and *FBLN5* in other brain regions. This variant also influenced the expression of several other genes across multiple tissues. Similarly, the rs7610 minor allele was linked to reduced *CHGA* expression in the hippocampus, lower *FBLN5* levels in the nucleus accumbens, and altered *ITPK1* and T*MEM251* expression in vascular and adipose tissues, respectively. Linked variants (rs9658631, rs9658634, rs7159323) were reported as eQTLs for *CHGA* in the hippocampus, with minor alleles associated with lower expression.

In addition, methylation QTL (mQTL) analysis from QTLbase identified several methylation associations for *CHGA* in blood (rs9658268 and rs7610: 6 mQTLs; rs9658234: 23 mQTLs; rs7159323: 18 mQTLs) and brain tissue (rs965863: 2 mQTLs; rs9658234: 4 mQTLs; rs7159323: 2 mQTLs). These SNPs also acted as mQTLs for other genes, particularly *ITPK1*, in both blood and brain.

## 4 Discussion

A central idea in Geroscience is that aging, age-related diseases, and longevity share common molecular and genetic mechanisms (Franceschi et al., 2018). Many genes linked to extended lifespan also influence disease risk, highlighting the complex, overlapping roles of genetic factors (Franceschi et al., 2020). Phenomena such as pleiotropy and epistasis further illustrate how individual genes can impact multiple traits or interact with others, offering insight into the biological pathways that connect aging, health, and longevity.

Within this context, the Chromogranin A (*CHGA*) gene stands out for its multifaceted and multifunctional nature. As the precursor to biologically active peptides, CHGA exerts pleiotropic effects across multiple systems, including the endocrine, neuroendocrine, cardiovascular, and immune systems (D’Amico et al., 2014; Corti et al., 2018), making it a particularly compelling candidate for exploring the genetic links between aging, health and longevity.

In this study, we adopted a case–control design based on two distinct age cohorts: older adults aged 65–85 years and long-lived individuals aged 86–107 years. Our objective was to evaluate whether selected SNPs in the *CHGA* gene are associated with survival at advanced age, and to assess their relationships with metabolic, clinical, and cognitive traits known to affect mortality risk.

The genetic associations we identified highlight the pleiotropic effects of *CHGA* locus on different traits.

Five of the nine *CHGA* SNPs examined (rs9658628- G/A, rs9658631- T/G, rs9658634-G/A, rs7159323- C/A, and rs7610- C/T) were significantly associated (FDR q < 0.05) with the odds of reaching an advanced age. The minor allele of rs9658628 was associated with reduced longevity, while the minor alleles of the other four variants conferred a protective effect. No evidence of effect modification by sex was observed. The associations between the tested SNPs and longevity were further validated through exploration of the TSI data and a bootstrap resampling analysis.

These findings support a role for *CHGA* genetic variation in modulating molecular and cellular pathways involved in aging and lifespan.

When we examined the effect of these SNPs in the older adult cohort on parameters associated with risk of death, we found several significant associations, the robustness of which was confirmed by bootstrap analyses stratified by sex. Moreover, the observed associations were not substantially modified by sex.

The rs9658628 A allele, which is associated with reduced longevity, was significantly associated with increased TyG index, a surrogate marker of insulin resistance, and increased risk of type 2 diabetes. These findings align with prior research demonstrating the role of CHGA and derived peptides, such as pancreastatin and catestatin, in regulating glucose metabolism, insulin sensitivity, and inflammation (Herold et al., 2021; Garg et al., 2023). Elevated levels of CHGA and pancreastatin are associated with type 2 diabetes, while the absence of these peptides reduces insulin resistance in mice (Kogawa et al., 2016; O’Connor et al., 2005; Friese et al., 2010). Based on our findings, we can speculate that the rs9658628 A-allele may reduce the likelihood of living a long life by predisposing individuals to insulin resistance and to an increased risk of diabetes.

Additionally, rs9658628 A-allele was associated with lower MMSE cognitive scores. This is notable considering that cognitive impairment is a known predictor of increased all-cause mortality in older adults (Glei et al., 2023; Wang et al., 2024). Of interest is the strong connection between insulin resistance, diabetes and cognitive decline in older people, which has been demonstrated by several studies (Liao, et al., 2025; Xie et al., 2022; You et al., 2021). Post-hoc adjustment for both diabetes status and MMSE scores in a fully adjusted model revealed that the rs9658628 A allele retained significance only for cognitive performance, suggesting that its association with diabetes may not be independent. Conversely, no correlation was observed between the TyG index and MMSE scores. Pleiotropic mechanisms, including horizontal pleiotropy, where the SNP affects multiple traits through independent pathways, and vertical pleiotropy, where the SNP influences one trait that subsequently impacts another, could account for the observed cross-phenotype associations.

Interestingly, despite its detrimental impact on longevity, the rs9658628-A allele was paradoxically associated with a reduced risk of CVD. Similar paradoxical effects have been reported in longevity studies (Tesi et al., 2021; Dato et al., 2021; Bellou and Escott-Price, 2023). They may indicate the presence of pleiotropic effects, possibly involving trade-offs between traits. For example, while a variant may confer vascular protection, it may also increase susceptibility to metabolic or neurodegenerative disease, potentially offsetting the overall benefit in terms of survival.

The pleiotropic nature of CHGA likely reflects the diverse and sometimes opposing biological activities of its proteolytic fragments. For instance, while pancreastatin exacerbates insulin resistance, catestatin enhances insulin sensitivity (Valicherla et al., 2013; Dasgupta et al., 2020; Mahata, et al., 2024). Other fragments, such as vasostatin-2 and catestatin, have been shown to be atheroprotective, with low levels associated with coronary artery disease (Chen et al., 2019; Lu, et al., 2012). However, catestatin levels are paradoxically elevated in heart failure, probably reflecting complex disease-specific mechanisms or compensatory responses (Kulpa, et al., 2025).

Functionally, rs9658628 is located ∼1 kb upstream of CHGA, suggesting a role in transcriptional regulation. It has been identified as an eQTL for *CHGA* in brain regions such as the cerebellum and prefrontal cortex, areas critical for cognitive function (Zhang et al., 2023; Xie et al., 2024; Jobson et al., 2021), providing a possible explanation for the observed association between rs9658628 and MMSE scores. Moreover, previous studies have reported CHGA downregulation in Alzheimer’s disease (Zhuang et al., 2025) and its presence in cerebellar unipolar brush cells (Nunzi and Mugnaini, 2009), further supporting its relevance to neurocognitive health. Additionally, rs9658628 appears to be a regulatory SNP or eQTL that regulates the expression of nearby genes, including *ITPK1* and *FBLN5* in the brain, potentially through mechanisms such as transcription factor binding, DNA methylation, and other epigenetic processes, as suggested by *in silico* analyses. *ITPK1* is key in inositol phosphate metabolism, which is crucial for intracellular signaling, neuronal calcium signaling and synaptic plasticity (Chamberlain et al., 2007; Chatree et al., 2020). *FBLN5* organizes and provides elasticity to the extracellular matrix, crucial for vascular integrity of elastic fibers in blood vessels (Cangemi et al., 2014). Its overexpression shows neuroprotection in rat brain injuries (Guo et al., 2016). These annotations can offer insights into the potential mediators and causal mechanisms accounting for the pleiotropic and heterogeneous effects of the variant across traits. Another notable finding of our study was the association of the rs7610 T allele with elevated DBP and, to a lesser extent, SBP. This variant has previously been linked to hypertension (Chen et al., 2008), where sex-dependent effects on its association with hypertension were reported. These effects may be mediated by reduced CHGA and catestatin levels via miR-107 regulation (Zhang et al., 2015). Despite its association with higher DBP, the rs7610 T allele was also linked to increased survival. This apparent contradiction may be explained by age-related shifts in blood pressure dynamics: while systolic pressure generally rises from young adulthood to old age, diastolic pressure peaks around middle age and then declines slightly (Franklin et al., 1997; Scuteri et al., 2014). Evidence shows that low DBP in older adults is more strongly linked to mortality risk than high DBP (Guichard et al., 2011), lending support to our findings.

The eQTL analysis revealed that the T allele is associated with increased *CHGA* expression in the pituitary gland, an organ playing a hormonal control of blood pressure (Whitworth, et al., 1983). The pituitary gland can produce CHGA (GTEx Consortium, 2020), and the CHGA levels and its derived peptides may both influence and be influenced by pituitary hormone secretion and blood pressure regulation. Also, for this variant eQTL effects on *ITPK1* and *FBLN5* were noted in vascular and brain tissues, respectively.

Regarding the three SNPs in strong LD (rs9658631, rs9658634, and rs7159323), while they were significantly associated with increased longevity, they showed no correlation with the specific endophenotypes studied. This may imply that their effects on survival may be mediated through other aging-related phenotypes not included in this analysis, highlighting the need for further research into alternative pathways of aging.

We acknowledge that the study presents some limitations. First, the case–control design does not establish causality; longitudinal data are needed to confirm the relationship between *CHGA* variants and longevity. Second, while we assessed several aging-related traits, unmeasured parameters may also mediate genetic effects on longevity. Third, the sample size was limited, potentially affecting statistical power. Consequently, although the associations between the genetic variants and clinical parameters reached nominal significance, they did not hold FDR correction. Nevertheless, given the biological plausibility and clinical relevance of the associations, these findings warrant further investigation and replication. Fourth, functional interpretations were based on public eQTL databases without experimental validation, which should be addressed in future studies. Finally, a key limitation is the absence of replication in large, independent cohorts. Although we explored allele frequencies and age-association in the 1,000 Genomes TSI population, the lack of phenotypic data restricted the scope of this external comparison. To partially address this, we performed a stratified bootstrap resampling analysis, which confirmed the robustness of our findings within the available sample. However, full replication in independent populations remains necessary.

In conclusion, our findings underscore the complex and pleiotropic role of *CHGA* gene variants on aging-related traits and longevity. While some variants influence metabolic and cognitive pathways known to affect mortality, others may act through mechanisms not captured in current models of aging. The paradoxical associations observed, for example, between longevity and CVD risk, reflect the multifaceted roles of CHGA and its derivatives in health and disease. These results reinforce the importance of CHGA as a regulatory hub in human aging and highlight the need for further functional studies to unravel the complex biological interactions involved.

## Data Availability

The raw data supporting the conclusions of this article will be made available by the authors, without undue reservation.
