# Supplementary material for: Chromogranin a gene variants influence survival at old age through pleiotropic effects on multiple age-related traits
Source: Front Aging. 2025 Sep 12;6:1625070. doi: 10.3389/fragi.2025.1625070 (PMC12463820; doi:10.3389/fragi.2025.1625070)
Supplement: Supplementary file 1 [file Supplementaryfile1.docx]

Supplementary Material

# Supplementary Figures and Tables


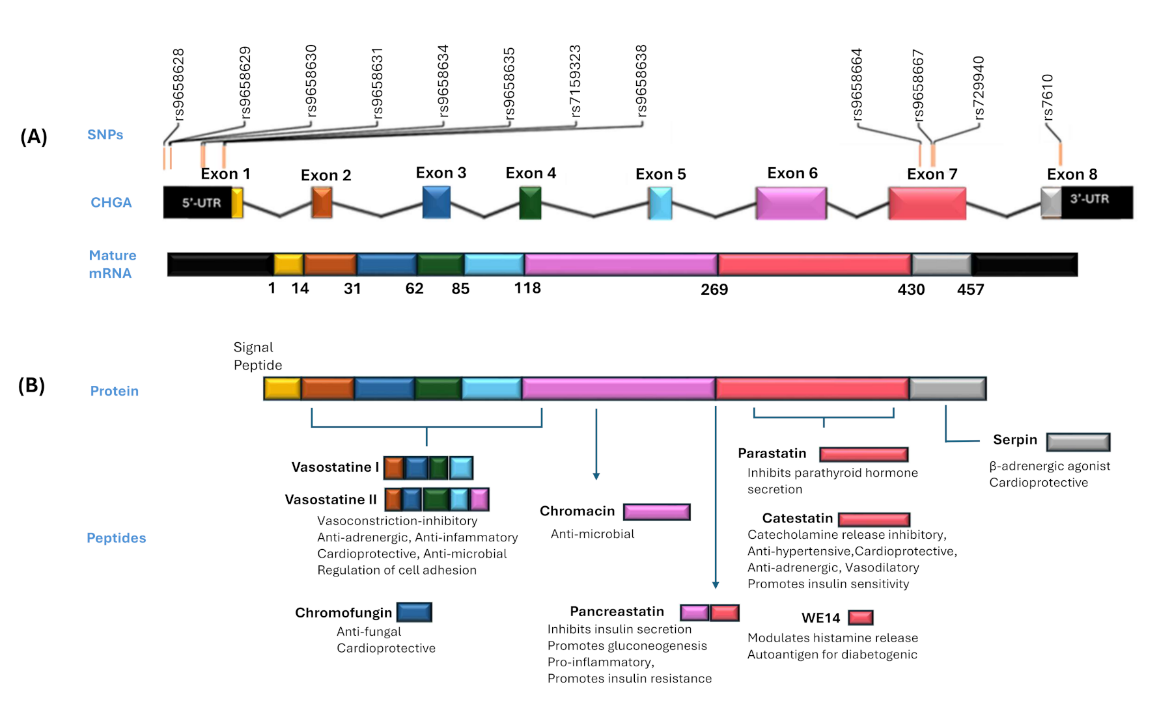


**Supplementary Figure S1.** Schematic of human *CHGA* structure and processing. (A) Structure of the *CHGA* gene, mature mRNA, and locations of the selected SNPs. The coloured blocks indicate exons, solid lines represent introns, black blocks represent upstream and downstream untranslated regions (UTRs). (B) Full-length CHGA protein and derived biologically active peptides and salient roles in physiological processes.

**Table S1.** Characteristics of the selected SNPs and the Minor Allele Frequencies (MAF) in the studied cohorts and Tuscany (TSI) population.

| SNP ID | Alleles  (major/minor) | Gene position^a^ | MAF  Older adults Long-lived Whole dataset TSI^b^ | | | |
| --- | --- | --- | --- | --- | --- | --- |
| rs9658628 | G/A | 1057bp 5' | 0.38 | 0.29 | 0.34 | 0.39 |
| rs9658629 | A/T | 969bp 5' | 0.10 | 0.11 | 0.10 | 0.07 |
| rs9658630 | T/C | 965bp 5' | Low call rate | | | 0.22 |
| rs9658631 | T/G | 939bp 5 | 0.21 | 0.31 | 0.26 | 0.22 |
| rs9658634 | G/A | 413bp 5' | 0.21 | 0.31 | 0.26 | 0.22 |
| rs9658635 | T/C | 366bp 5' | 0.20 | 0.19 | 0.19 | 0.15 |
| rs7159323 | C/A | 40bp 5 | 0.21 | 0.30 | 0.25 | 0.22 |
| rs9658638 | C/T | 8bp 5' | 0.10 | 0.10 | 0.10 | 0.08 |
| rs9658664 | G/A | Exon7  (G297S) | No HWE | | | 0.01 |
| rs9658667 | G/A | Exon7  (G364S) | Low call rate | | | 0.03 |
| rs729940 | C/T | Exon 7 (R381W) | 0.11 | 0.11 | 0.11 | 0.15 |
| rs7610 | C/T | Exon 8/3′-UTR | 0.30 | 0.39 | 0.34 | 0.29 |

^a^ Gene positions of the SNPs are based on RefSeq gene annotation database.

^b^ MAF according to 1000 Genomes for the TSI population (The 1000 Genomes Project Consortium, 2015)

**Table S2**. Summary of functional annotations on the *CHGA* gene of the significant SNPs using various bioinformatic tools, based on the GRCh38/hg38 genome assembly.

|  | Alleles | Regulatory potential | | Expression levels | | Epigenetic regulation | |
| --- | --- | --- | --- | --- | --- | --- | --- |
|  |  |  | | eQTL | | hQTL | mQTL |
| SNP ID |  | FuncPred | Regulome | GTEx | QTLbase | QTLbase | QTLbase |
| rs9658628 | G/A | 0.150 | 5 | Brain, Cerebellum | Brain Prefrontal Cortex | **_** | Blood,  Brain-Prefrontal Cortex |
| rs9658631 | G/T | 0.150 | 1f | **_** | Brain, Hippocampus | **_** | Blood,  Brain-Prefrontal Cortex,  Blood T-cell CD4+ naive |
| rs9658634 | A/G | 0.150 | 1b | **_** | Brain, Hippocampus | **_** | Blood,  Brain-Prefrontal Cortex, Kidney |
| rs7159323 | A/C | 1.000 | 1f | **_** | Hippocampus | **_** | Blood,  Brain-Prefrontal Cortex |
| rs7610 | T/C | 0.264 | 1f | Pituitary gland | Hippocampus | **_** | Blood,  Brain-Prefrontal Cortex |

Annotations include regulatory potential scores, predicted functional consequences, expression quantitative trait loci (eQTL), histone modification QTLs (hQTL), DNA methylation QTLs (mQTL). The regulatory potential score obtained by Func Pred ranges from 0 to 1 and it provides a probabilistic assessment of SNP functionality based on the integration of different tools in SNPinfo Web Server; the regulatory potential score by RegulomeDB classifies variants from 1a (most likely to affect regulation) to 7 (least likely or no data available). Data on eQTL, hQTL and mQTL are reported by indicating the most significant tissue. Missing data are indicated by a dash, reflecting the absence of significant signals or unavailable information in the queried datasets.

**Table S3**. Results of eQTL (expression Quantitative Trait Loci), mQTL (methylation QTL), and hQTL (histone modification QTL) analyses for the analysed SNPs, based on data from QTLbase.

| SNPID | QTL type | Molecular trait | Effective Allele | Effect size | P-value | FDR | Tissue |
| --- | --- | --- | --- | --- | --- | --- | --- |
| rs9658628 | eQTL | FBLN5 | NA | NA | 0.001 | NA | Brain |
| rs9658628 | eQTL | CHGA | NA | NA | 0.010 | NA | Brain Prefrontal Cortex |
| rs9658628 | eQTL | CPSF2 | NA | -0.332 | 0.005 | 0.033 | Blood-T cell CD4+ naive |
| rs9658628 | eQTL | GOLGA5 | NA | NA | 2.460e-4 | NA | Stem cell-iPSC |
| rs9658628 | eQTL | LINC02287 | NA | -0.363 | 0.005 | NA | Kidney |
| rs9658628 | eQTL | ITPK1 | NA | NA | 0.001 | NA | Brain |
| rs9658628 | eQTL | ITPK1 | G | NA | 0.008 | 0.198 | Blood-Neutrophils CD16+ |
| rs9658628 | eQTL | TMEM251 | NA | -0.278 | 0.010 | 0.587 | Blood-Neutrophils CD16+ |
| rs9658628 | eQTL | BTBD7 | NA | -0.298 | 0.006 | 0.053 | Blood-Monocytes CD14+ |
| rs9658628 | mQTL | cg13729548-ITPK1 | A | 0.027 | 7.790e-25 | NA | Blood |
| rs9658628 | mQTL | cg08087268-ITPK1 | A | 0.020 | 2.250e-28 | NA | Blood |
| rs9658628 | mQTL | cg23013853-CHGA | A | 0.288 | 2.400e-9 | 0.002 | Blood |
| rs9658628 | mQTL | cg23013853-CHGA | NA | NA | 1.920e-9 | NA | Brain-Prefrontal Cortex |
| rs9658628 | hQTL | H3K4me1-LGMN | A | 0.050 | 0.006 | 0.610 | Blood-T cell CD4+ naive |
| rs9658631 | eQTL | CHGA | T | -0.220 | 1.720e-4 | NA | Brain |
| rs9658631 | eQTL | CHGA | G | -0.159 | 1.250e-5 | 0.002 | Brain Hippocampus |
| rs9658631 | eQTL | FBLN5 | NA | NA | 9.430e-5 | NA | Brain |
| rs9658631 | eQTL | FBLN5 | T | 0.252 | 7.790e-6 | NA | Brain-Nucleus Accumbens |
| rs9658631 | mQTL | cg23013853-CHGA | G | -0.887 | 7.420e-89 | 1.730e-80 | Blood |
| rs9658631 | mQTL | cg23013853-CHGA | G | -0.042 | 1.510e-29 | 3.670e-27 | Brain-Prefrontal Cortex |
| rs9658631 | mQTL | cg23013853-CHGA | G | 1.195 | 8.250e-19 | 3.220e-16 | Blood-T cell CD4+ naive |
| rs9658631 | mQTL | cg00813752-CHGA | G | -0.711 | 1.500e-45 | 1.350e-37 | Blood |
| rs9658631 | mQTL | cg00813752-CHGA | G | -0.036 | 2.040e-20 | 2.420e-18 | Brain-Prefrontal Cortex |
| rs9658631 | mQTL | cg16290737-CHGA | G | -0.822 | 7.680e-73 | 6.530e-65 | Blood |
| rs9658631 | hQTL | H3K4me1-LGMN | T | -0.092 | 2.850e-4 | 0.067 | Blood-Monocytes CD14+ |
| rs9658634 | eQTL | ITPK1 | A | NA | 3.760e-13 | NA | Blood |
| rs9658634 | eQTL | ITPK1 | NA | 0.422 | 0.002 | 0.169 | Blood-T cell CD4+ naive |
| rs9658634 | eQTL | FBLN5 | G | 0.251 | 8.110e-6 | NA | Brain-Nucleus Accumbens |
| rs9658634 | eQTL | FBLN5 | NA | NA | 1.720e-4 | NA | Brain |
| rs9658634 | eQTL | CHGA | G | -0.210 | 2.390e-4 | NA | Brain |
| rs9658634 | eQTL | CHGA | A | -0.153 | 2.490e-5 | 0.004 | Brain-Hippocampus |
| rs9658634 | eQTL | LINC02287 | A | 0.570 | 5.590e-4 | 0.032 | Blood-T cell CD4+ naive |
| rs9658634 | eQTL | LGMN | A | -0.107 | 0.003 | 0.102 | Blood-T cell CD4+ naive |
| rs9658634 | mQTL | cg23013853-CHGA | A | -0.286 | 2.090e-26 | NA | Blood |
| rs9658634 | mQTL | cg23013853-CHGA | A | -0.043 | 1.380e-30 | 3.620e-28 | Brain-Prefrontal Cortex |
| rs9658634 | mQTL | cg00813752-CHGA | A | -0.190 | 1.900e-15 | NA | Blood |
| rs9658634 | mQTL | cg00813752-CHGA | A | -0.037 | 8.640e-22 | 1.150e-19 | Brain-Prefrontal Cortex |
| rs9658634 | mQTL | cg16290737-CHGA | A | -0.228 | 2.030e-17 | NA | Blood |
| rs9658634 | mQTL | cg16290737-CHGA | A | -0.031 | 2.870e-21 | 3.650e-19 | Brain-Prefrontal Cortex |
| rs9658634 | mQTL | cg16290737-CHGA | G | 0.480 | 2.380e-16 | NA | Kidney |
| rs9658634 | hQTL | H3K4me1-LGMN | G | -0.092 | 2.850e-4 | 0.067 | Blood-Monocytes CD14+ |
| rs7159323 | eQTL | FBLN5 | NA | NA | 6.660e-5 | NA | Brain |
| rs7159323 | eQTL | FBLN5 | C | 0.258 | 6.370e-6 | NA | Brain-Nucleus Accumbens |
| rs7159323 | eQTL | LINC02287 | NA | NA | 0.006 | NA | Kidney |
| rs7159323 | eQTL | CHGA | A | -0.180 | 5.020e-5 | 0.008 | Brain-Hippocampus |
| rs7159323 | mQTL | cg16290737-CHGA | A | -0.792 | 5.550e-69 | 3.480e-61 | Blood |
| rs7159323 | mQTL | cg23013853-CHGA | A | -0.837 | 8.440e-80 | 1.080e-71 | Blood |
| rs7159323 | mQTL | cg23013853-CHGA | A | -0.049 | 1.890e-31 | 5.290e-29 | Brain-Prefrontal Cortex |
| rs7159323 | mQTL | cg00813752-CHGA | A | -0.045 | 2.370e-25 | 4.210e-23 | Brain-Prefrontal Cortex |
| rs7159323 | hQTL | H3K4me1-LGMN | C | -0.087 | 7.440e-4 | 0.128 | Blood-Monocytes CD14+ |
| rs7610 | eQTL | CHGA | T | -0.188 | 2.050e-5 | 0.003 | Brain- Hippocampus |
| rs7610 | eQTL | ITPK1 | C | 0.139 | 1.100e-5 | NA | Artery-Aorta |
| rs7610 | eQTL | ITPK1 | C | 0.087 | 7.170e-5 | NA | Artery-Tibial |
| rs7610 | eQTL | FBLN5 | NA | NA | 3.080e-4 | NA | Brain |
| rs7610 | eQTL | FBLN5 | C | 0.224 | 2.600e-5 | NA | Brain-Nucleus Accumbens |
| rs7610 | eQTL | TMEM251 | C | 0.195 | 0.009 | NA | Adipose Tissue |
| rs7610 | mQTL | cg23013853-CHGA | T | -0.713 | 2.230e-62 | 1.220e-54 | Blood |
| rs7610 | mQTL | cg23013853-CHGA | T | -0.038 | 5.330e-19 | 5.570e-17 | Brain-Prefrontal Cortex |
| rs7610 | mQTL | cg00813752-CHGA | T | -0.591 | 2.840e-36 | 8.440e-29 | Blood |
| rs7610 | mQTL | cg16290737-CHGA | T | -0.638 | 3.460e-48 | 9.380e-41 | Blood |
| rs7610 | mQTL | cg16290737-CHGA | T | -0.028 | 2.550e-14 | 1.680e-12 | Brain-Prefrontal Cortex |
| rs7610 | mQTL | cg23920975-ITPK1 | NA | NA | 2.120e-18 | NA | Brain-Prefrontal Cortex |
| rs7610 | mQTL | cg08087268-ITPK1 | T | -0.018 | 9.940e-29 | NA | Blood |
| rs7610 | hQTL | H3K4me1-LGMN | C | -0.077 | 0.003 | 0.252 | Blood-Monocytes CD14+ |
| rs7610 | hQTL | H3K27ac-GOLGA5 | C | 0.062 | 0.010 | 0.061 | Blood-Neutrophils CD16+ |

Effective Allele refers to the allele of the SNP associated with the reported effect size on the molecular trait. Effect size represents the magnitude and the direction of the change in the molecular trait associated with each copy of the effective allele. Only results with a p-value lower than 0.010 were reported.
